# Prevalence and Characteristics of Borderline Intellectual Functioning in a Cohort of Patients With Polysubstance Use Disorder

**DOI:** 10.3389/fpsyt.2021.651028

**Published:** 2021-07-14

**Authors:** Jens Hetland, Kirsten J. Braatveit, Egon Hagen, Astri J. Lundervold, Aleksander H. Erga

**Affiliations:** ^1^KORFOR - Center of Alcohol and Drug Research, Division of Psychiatry, Stavanger University Hospital, Stavanger, Norway; ^2^Department of Biological and Medical Psychology, University of Bergen, Bergen, Norway; ^3^Department of Research and Innovation, Helse Fonna HF, Haugesund, Norway; ^4^Haugaland A-senter, Blue Cross Norway, Haugesund, Norway; ^5^The Norwegian Center for Movement Disorders, Stavanger University Hospital, Stavanger, Norway

**Keywords:** polysubstance use disorder, borderline intellectual functioning, symptom check list-90-R, satisfaction with life scale, intelligence quotient, prevalence, substance use disorder

## Abstract

**Objective:** To determine the prevalence and associated demographic and clinical features of borderline intellectual functioning (BIF) among individuals with polysubstance use disorder (pSUD).

**Methods:** We applied a cross-sectional analytical design to data from the Norwegian STAYER study (*n* = 162), a cohort study of patients with a pSUD from the Stavanger University hospital catchment area. We used Wechsler Abbreviated Scale of Intelligence Full Scale IQ (FSIQ) to define BIF (FSIQ = 70–85) and non-BIF (FSIQ = >85) and collected demographic and clinical data using semi-structured interviews and self-reports on the Symptom Checklist 90-Revised (SCL-90-R) and the Satisfaction With Life Scale (SWLS).

**Results:** The prevalence of BIF was 18% in the present study. The presence of BIF was associated with higher SCL-90-R GSI scores than in the non-BIF group. There were no significant differences between the BIF and non-BIF groups regarding age, gender, participation in meaningful daily activity, years of work experience, years of education, satisfaction with life, level of care, treatment attempts, age at substance-use onset, years of substance use, history of injecting drugs, or age of onset of injecting drugs.

**Conclusion:** The present study confirmed a higher prevalence of BIF among patients with pSUD than expected from the distribution of IQ scores in a general population. Elevated SCL-90-R GSI scores suggested that BIF is associated with increased psychological distress in patients receiving treatment for pSUD. Further studies on this association, and its effect on treatment procedure and outcomes are strongly warranted.

## Introduction

Intellectual functioning in patients with substance use and abuse has received increased attention during the last decade (1, 2). This follows the fact that intellectual functioning (e.g., reasoning, planning, problem solving, judgement, and abstract thinking) is a core predictor of a variety of life outcomes, with the most severe impairments observed in patients with an intelligence quotient (IQ) two standard deviations below the population mean (IQ < 70) (3–5). In the present study, we focused on the impact of borderline intellectual functioning (BIF), which is defined as an intelligence quotient ranging between one and two standard deviations below the population mean (IQ = 70–85). Based on previous studies, we know that adults with BIF have an increased vulnerability for developing psychiatric disorders, including a substance use disorder (SUD) (2, 6–12). Assessment of intellectual function should therefore be considered an important component of clinical examination and treatment planning of SUDs.

According to the normal distribution of IQ scores (Bell Curve), approximately 13.6% of individuals in the general population would be allocated to a subgroup defined with BIF, with elevated rates commonly observed in clinical populations (13). Nevertheless, the frequency estimates within clinical groups are uncertain because of methodological differences between studies (ascertainment biases, the choice of diagnostic tools, service configurations, and entry criteria). In addition, there is a historical lack of terminological consensus and classification of BIF (14, 15) and non-agreed-upon diagnostic criteria in diagnostic manuals like the DSM-V and ICD-10 (16, 17). Nevertheless, studies have shown that individuals with BIF exhibit difficulties in several aspects of life, that these difficulties may occur at a similar level as for individuals with a diagnoses of intellectual disability (ID), and that individuals with BIF may need targeted support (1, 4, 6, 9, 10, 14, 18–21).

Individuals with BIF may not only be severely impaired; they are also less likely to receive adequate treatment for mental health issues, less likely to receive psychotherapy, and more likely to be treated with psychotropic medication than individuals with mental health problems in the general population (10, 22). This is obviously the case in individuals with co-occurring BIF and SUD; they tend to show adverse rehabilitation outcomes when offered mainstream SUD treatment, because of factors such as reduced disposition to change and desire for help (23, 24), lower treatment compliance (25), high drop-out rate (26–28), relapse during treatment (29), and negative treatment experiences (30). Therefore, it is alarming to realize that impaired intellectual functioning is often overlooked in treatment programs for patients with SUD, even though it can be a key clinical factor in predicting treatment needs and prognosis (24, 29, 31–34).

There is a dearth of research on BIF in general, and BIF in SUD populations in particular. When included in studies, BIF is typically classified broadly as mild-to-borderline intellectual disability (MBID) with IQ ranging between 50 and 85, or treated as a control group (4). The major thrust of research on the co-occurrence of BIF and SUD originate from the field of ID services and target substance use in individuals with a known ID diagnosis. Subsequently, findings are mainly published in journals in that field, rather than in journals in the field of medical addiction (2). Initiatives to develop a framework around the clinical and adaptive needs of patients with co-occurring SUD and BIF have been sporadic and uncoordinated (14).

Studies examining the prevalence rates of BIF in SUD populations are scarce, and their prevalence rates vary considerably. Braatveit et al. found the prevalence rate of BIF among patients with SUD to be 23% (29), and Luteijn et al. reported a MBID prevalence rate of 39% (24). At the other end of the scale, VanDerNagel et al. reported a prevalence estimate as low as 3% (35). Furthermore, prevalence data for BIF and MBID are difficult to compare because of lack of consensus on terminology, differences in group characteristics, levels of disability, treatment settings, comorbid psychiatric disorders, and definition and scope of substance use (2, 13, 36). Taken together, studies of BIF based on standard instruments in well-characterized cohorts of patients with SUD are obviously warranted.

The lack of epidemiological data and findings showing that BIF may be vital for the broader understanding and treatment of patients with SUD motivated the present study to investigate the prevalence and characteristics of patients with BIF in a typical group of individuals receiving treatment for polysubstance use disorder (pSUD). Polysubstance use is common in both clinical, and population samples (37, 38). Moreover, polysubstance use patterns is frequent in patients seeking treatment for mono-substance disorders (39–43). In this context, pSUD refers to the use of multiple substances as part of a pattern of problematic substance use, in which the patient meets criteria for SUD for some, but not necessarily all substances used (44). Compared with mono-substance users, polysubstance users have an earlier onset of substance use (45), are younger (37), have higher levels of psychological distress and personality disorders (45–50), more persistent cognitive impairments (51), and poorer social adjustment (37, 46, 48, 52). Studies suggest that these characteristics are associated with increased risk of dropout and relapse (27, 53–57). Thus, patients with pSUD may have a more severe clinical profile than patients with mono-substance use and consequently pose a challenge for SUD-treatment services and the mental health care system (46, 53, 58, 59).

The aim of the present study is twofold: (1) to provide a prevalence estimate of BIF in patients with pSUD receiving mainstream SUD treatment (2) to investigate clinical and demographic features in subgroups of patients with and without co-existing BIF.

## Materials and Methods

### Study Design and Patient Characteristics

The study used data from the Stavanger Study of Trajectories of Addiction (STAYER), an ongoing, prospective, longitudinal cohort study of the neurocognitive, psychological and social recovery in patients with polysubstance use who started a new treatment sequence in the Stavanger University Hospital catchment area (60, 61). See Andersson et al. (54) for more details regarding the structure of Norwegian SUD-treatment. To be eligible for specialized treatment for SUDs within the Norwegian public health service, patients must meet the criteria for a F1x.1 (harmful use) or F1x.2 (dependency syndrome) diagnosis, as defined by the ICD-10 (17). We performed baseline assessment after 2 weeks of abstinence, in an attempt to minimize contamination from drug withdrawal and the acute neurotoxic effects from psychoactive substances (62). Trained research personnel of the STAYER research group collected all data. In the present study, polysubstance users were defined as patients with SUD who reported the use of multiple substances within the last year before inclusion. The project was approved by the Regional Ethics Committee (REK 2011/1877) and conducted according to its guidelines and those of the Helsinki Declaration (1975). All participants provided signed informed consent.

### Participants

A total of 208 patients were recruited consecutively at convenience from 10 outpatient and residential treatment facilities within the Stavanger University Hospital catchment area between March 2012 and January 2016. All patients had been voluntary admitted for SUD-treatment.

Patients were included if they (1) signed a written informed consent, (2) were enrolled in a new rehabilitation sequence by the substance use treatment service, (3) reported use of multiple substances within the last year before inclusion, and (4) were 16 years or above. Patients received a compensation of NOK 400 for their time at the baseline testing. Of the 208 patients in the STAYER cohort, 44 patients were excluded from the present study because of mono-substance use (alcohol *N* = 35, cannabis *N* = 1) or lack of substance-related disorders (e.g., gambling *N* = 8). We excluded one case because of missing IQ scores and one case because of an IQ score <70; thus, the remaining sample of patients with pSUD comprised 162 individuals.

### Assessment

We obtained demographic, neurocognitive, psychological, and social-functioning data using semi-structured interviews, cognitive tests, and self-reported measures at the baseline assessment. We used a preliminary version of the National Quality Register for Substance Abuse (KVARUS) (63), a semi-structured interview to obtain information on the type of substance intake, initial age at use, treatment and work history, and educational, vocational, and social adjustment.

#### Wechsler Abbreviated Scale of Intelligence

Wechsler Abbreviated Scale of Intelligence (WASI) (64) was used to assess intellectual function. WASI was created to establish a brief and reliable estimate of intellectual functioning and comprises four subtests, i.e., two verbal measures of crystalized intelligence (Vocabulary and Similarities), which yield a verbal intelligence quotient (VIQ), and two non-verbal tests of fluent intelligence (Block Design and Matrix Reasoning), which yield a performance intelligence quotient (PIQ). BIF was defined as a WASI Full-scale IQ (FSIQ) ranging between 70 and 85, and non-BIF was defined as a FSIQ > 85.

#### Satisfaction With Life Scale

Satisfaction with life was assessed using the Satisfaction With Life Scale (SWLS) (65). SWLS is a self-report questionnaire comprising five items to measure the respondent's global life satisfaction with a seven-point Likert-type format (ranging from 1-strongly disagree to 7-strongly agree). SWLS has demonstrated excellent psychometric characteristics (66) and also validated for individuals with ID (Cronbach's alpha = 0.79) (67). A score of 20 represents a neutral point on the scale; scores between 5 and 9 indicate dissatisfaction with life, while scores ranging between 31 and 35 indicate that the respondent is very satisfied with life (66).

#### Symptom Checklist 90-Revised

We used the Symptom Checklist 90-Revised (SCL-90-R), which is a 90-item self-report measure (68) assessing psychological symptoms and distress. SCL-90-R is widely used in clinical practice and research, and validated for patients with SUD and individuals with ID (68–70). Items are rated on a five-point Likert scale indicating the degree of distress, ranging from 0 (not at all) to 4 (severely) during the 7 previous days. The checklist comprises nine symptom dimension subscales: Somatization, Obsessive–Compulsive Disorder, Interpersonal Sensitivity, Depression, Anxiety, Hostility, Phobic Anxiety, Paranoid Ideation, and Psychoticism, in addition to a global severity index (GSI), which was used here as a measure of psychological distress.

### Statistics

The statistical software package SPSS version 26 (IBM Corp., released 2016) was used for all statistical analyses. Statistical significance was set at *P* < 0.05, and assumptions of normality evaluated based on Q–Q plots and by inspecting the residuals. A frequency analysis was run for the BIF and non-BIF groups. Independent-sample *t*-tests were performed to evaluate differences between-group means, and the chi-squared test of independence was used in case of categorical variables.

Because of an association between BIF status and SCL-90-R GSI score, we performed additional *post hoc* analyses to explore this association. As a result of the modest size of the BIF group, we opted not to use BIF status as a dependent variable in logistic regression analyses because of the risk of overfitting the regression model (71). Instead, we performed a multiple regression analysis (forward selection) with SCL-90-R GSI score as the dependent variable and BIF status, age, gender, years of education, age of onset of substance use, history of injecting drugs, and SWLS sum score as independent variables.

## Results

Among the 162 participants included in the analyses, 29 (17.9%) were classified as having BIF. Table 1 shows the demographic and clinical features in the total sample and stratified according to intellectual functioning (i.e., the BIF and non-BIF group). Participants in the BIF group (M = 1.4, SD = 0.8) exhibited significantly higher SCL-90-R GSI scores than the non- BIF group [M = 1.1, SD = 0.6; *t*_(160)_ = 2.5, *p* < 0.05], indicating a higher degree of self-reported psychological distress in the former group. No further significant differences were detected between the BIF and non-BIF groups on any demographic or clinical feature.

**Table 1:** Demographic and clinical features of the present sample stratified according to intellectual functioning.

	**Total sample**		**BIF (*****n*** **= 29)**		**Non-BIF (*****n*** **= 133)**		**Statistics**	
	***n***	**Mean (SD)/*n* (%)**	***n***	**Mean (SD)**	***n***	**Mean (SD)**	***t*(df)/Value (df)**	**Cohen's d**	***P*-value**
Age	162	27.6 (7.5)	29	26.1 (8.4)	133	27.9 (7.3)	−1.22 (160)	0.24	0.225
Male gender^*^	162	106 (65.4)	18 (62.1)		88 (66.2)		−0.18 (1)		0.674
Income from work or other meaningful daily activity^*^	162	101 (62.3)	17 (58.6)		84 (63.2)		0.21 (1)		0.648
Years of work experience	146^a^	5.6 (5.8)	26	4.0 (4.1)	120	5.9 (6.1)	−1.51 (144)	0.36	0.134
Education, years	162	11.6 (1.7)	29	11.2 (1.7)	133	11.7 (1.7)	−1.18 (160)	0.24	0.239
Treatment attempts	162	1.6 (2.4)	29	1.5 (2.0)	133	1.6 (2.4)	−0.29 (160)	0.06	0.776
In-patient^*^	161^a^	95 (58.6)	20 (71.4)		75 (56.4)		2.16 (1)		0.141
SCL-90-R GSI	162	1.1 (0.7)	29	1.42 (0.8)	133	1.1 (0.6)	2.48 (160)	0.46	0.014
SWLS sum score	162	15.4 (6.3)	29	14.8 (6.1)	133	15.5 (6.4)	−0.57 (160)	0.12	0.569
Age of drug debut	160^a^	13.1 (2.1)	29	12.7 (1.7)	131	13.1 (2.2)	−0.95 (158)	0.21	0.343
Years of drug use	160^a^	14.5 (7.5)	29	13.3 (8.1)	131	14.8 (7.4)	−0.95 (158)	0.18	0.343
Injected drugs^*^	161^a^	98 (60.5)	15 (51.7)		83 (62.9)		1.24 (1)		0.265
Age at first use of injected drugs	98^b^	19.7 (5.0)	15	18.2 (5.8)	83	20.0 (4.8)	−1.29 (96)	0.36	0.202

* *Chi-squared test of independence*.

a *Numbers lower than 162 are caused by missing data*.

b *Participants with a history of injecting drugs*.

**Figure 1:**
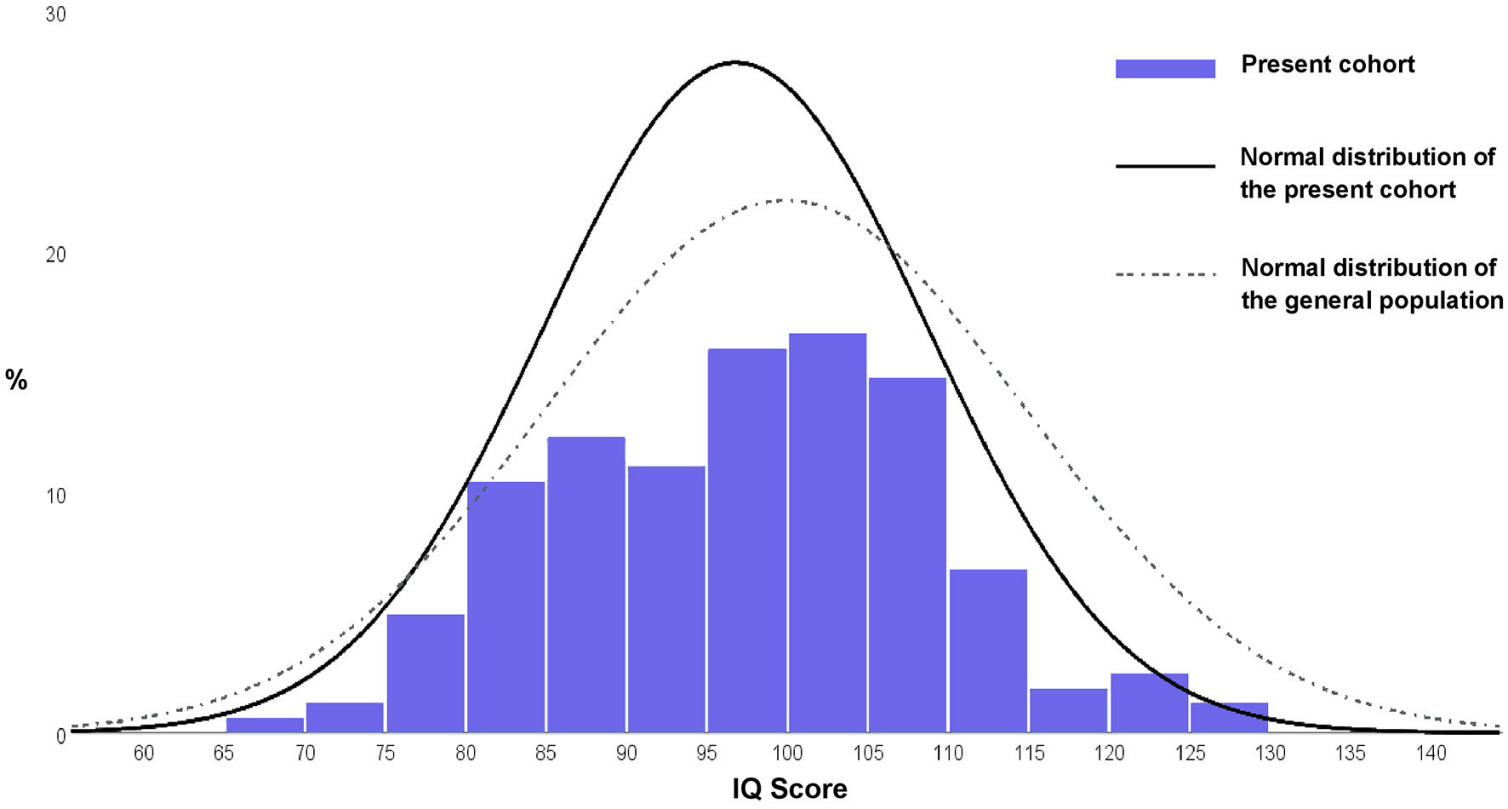
Histogram of the distribution of IQ scores in the present cohort compared with the theoretical distribution of the general population. One participant with IQ < 70 was included in the histogram.

Figure 1 shows that the distribution of IQ scores in the present cohort was comparable to the expected distribution in the general population, with a small shift toward the lower end of the scale.

Table 2 lists the WASI scores in the total sample and within the two groups. The mean WASI FSIQ in this BIF group was 80.3 (SD = 3.8, 95% CI = 78.8–81.7), whereas the mean WASI FSIQ was 100.8 (SD = 9.4, 95% CI = 99.1–102.4) in the non-BIF group.

**Table 2:** WASI scores in the total sample stratified according to intellectual functioning.

	**Total sample**		**BIF (*****n*** **= 29)**		**Non-BIF (*****n*** **= 133)**		**Statistics**		
	***n***	**Mean (SD)**	***n***	**Mean (SD)**	***n***	**Mean (SD)**	***t*(df)**	**d**	***P* value**
WASI FSIQ	162	97.1 (11.7)	29	80.3 (3.8)	133	100.8 (9.4)	−11.5 (160)	2.85	<0.001
WASI VIQ	162	95.1 (12.7)	29	82.5 (8.2)	133	97.8 (11.8)	−6.6 (160)	1.51	<0.001
WASI PIQ	162	99.9 (13.2)	29	82.0 (8.1)	133	103.8 (10.7)	−10.4 (160)	2.30	<0.001

A multiple regression analysis using the SPSS' forward selection algorithm was computed to further investigate the association between the presence of BIF and the SCL-90-R GSI scores. The SCL-90-R GSI scores were included as the dependent variable and the BIF status as well as age, gender, years of education, age of onset of substance use, history of injecting drugs, and SWLS sum score as independent variables. This procedure yielded a significant regression equation F_(3,156)_ = 14.882, *P* < 0.001; *R*^2^ = 0.223), leaving BIF status as well as age, and SWLS sum score as significant predictors of the SCL-90-R GSI scores (see Table 3 for details).

**Table 3:** Summary of the regression analysis with SCL-90-R GSI as dependent and BIF status, age, and SWLS sum as independent variables.

**Variable**	**B**	**95% CI**	**β**	***t***	***p***
(Constant)	2.533	[2.082, 2.983]		11.106	<0.001
SWLS Sum score	−0.039	[−0.054, −0.024]	−0.369	−5.219	<0.001
Age	−0.021	[−0.033, −0.008]	−0.233	−3.283	0.001
BIF-status	−0.256	[−0.499, −0.014]	−0.148	−2.086	0.039

*BIF status is coded as 0 for BIF and as 1 for non-BIF*.

*CI, confidence interval for B*.

## Discussion

The prevalence rate of BIF in patients with polysubstance use was 18% in the present study. There were few statistically significant differences between the BIF and non-BIF groups regarding demographic and clinical features. However, patients with BIF had significantly elevated SCL-90-R GSI scores, indicating a higher degree of psychological distress compared with the non-BIF group. A regression analysis confirmed the importance of BIF status, even when controlling for a range of demographic and clinical data.

The prevalence rate of BIF found in the current study was higher than that observed in the general population, but still somewhat lower than reported by some previous studies of patients selected from in-patient SUD populations (24, 29). However, the sample included in the study of Luteijn et al. (24) was selected from a forensic unit and gauged the prevalence rate of MBID, not BIF. Although it may be tempting to hypothesize that patients receiving in-patient treatment have more impaired intellectual functioning compared to patients receiving out-patient treatment, the results of the current study do not support this notion, as there were no significant differences in the prevalence rate of BIF between these two groups. The prevalence rate of BIF found in the present study was indeed higher than the 3% identified by VanDerNagel et al. (35). However, those authors relied on the identification of individuals with BIF through a review of caseloads and patient records. Because of the low recognition of MBID/BIF, those findings are expected to provide underestimations compared with the results of studies including direct assessment of intellectual functioning.

The regression model indicated independent negative associations between the independent variables SWLS sum score, age, and BIF-status and SCL-90-R GSI score among patients with pSUD. The association between SWLS sum score and SCL-90-R GSI score was expected, given the conceptual similarities between psychological well-being and life satisfaction in human functioning. In addition, age was negatively associated with SCL-90-R GSI scores, a finding that was expected based on previous studies (44, 72). A strong association between BIF and an elevated SCL-90-R GSI score among patients suffering from pSUD is a main finding of the present study. This finding is in accordance with previous studies reporting associations between psychological distress and impaired intellectual functioning (19, 73–76). Although causality of the association between SCL-90-R GSI score and BIF status in the present study is unknown, several direct and indirect paths may be suggested.

Individuals with impaired intellectual functioning may be susceptible to the development of psychological ill-health and impaired social adjustment due to reduced capacity for problem-solving, flexible adjustment and stress tolerance (77). Conversely, psychiatric disorders may induce temporary state-specific neurocognitive disruptions impairing cognitive performance (78–80). Finally, the selected measures may not reflect disparities in latent cognitive abilities as psychological distress may impede test performance indirectly through lack of performance motivation, low self-efficacy and increased engagement in distracting worrisome thoughts or task-irrelevant cognition.

The use of an IQ criterion in the diagnosis of ID is thought to reflect a relationship between intellectual and everyday functioning, and most studies identify borderline intellectual disability solely from intellectual functioning measures, i.e., BIF (29). While the current study found disparities in the associated clinical features between the BIF and non-BIF patients with pSUD, the differences were primarily reserved to the SCL-90-R GSI score. Surprisingly, the findings thus did not support the presence of a more global impairment in BIF compared to non-BIF patients with pSUD. e.g., educational attainment is typically shown to be associated with higher intellectual functioning (81–83). However, to access specialized treatment for SUDs within the Norwegian public health service, patients must exhibit severely debilitating substance use. Furthermore, both the BIF, and non-BIF groups share approximately the same early onset of substance use (13 years). Both early onset and subsequent severe substance use likely attenuate the predictive value of IQ by exerting a major detrimental influence on scholastic performance (84), attendance (85), drop out (86–89), and overall social adjustment.

The present study used the classification of BIF rather than borderline intellectual disability, as the latter relies on additional measures of adaptive functioning and onset before 18 years of age. In addition, several studies investigated the clinical features of co-occurring BIF and SUD by combining the IQ ranges of BIF and mild ID (2, 24, 35, 90, 91). The risk factors and associations identified in these studies may result from the inclusion of a proportion of individuals with ID. Alternatively, our results may be used to argue that intellectual functioning, as measured by WASI or otherwise, may be less useful when accounting for differences in clinical features and everyday functioning in patients with SUD.

### Strengths and Limitations

The current cohort was recruited from a multitude of specialized and diversified SUD rehabilitation services including both in- and out-patient units targeting different patient groups with regard to type and severity of comorbid psychiatric disorders, the severity of substance use, and degree of social adjustment and functioning, as well as the stage of the rehabilitation process. The universal access to health care in Norway allows the collection of a more comprehensive sample relative to countries where care is privatized and costly. Thus, the findings of the current study cannot necessarily be generalized to a specific clinical population (e.g., in-patients), but do elucidate the general state of intellectual functioning and associated clinical features among patients with pSUD.

Most previous studies investigated the clinical features of individuals with substance use among patients already identified as having ID (IQ < 70) or MBID (IQ = 50–85) (2). To the authors knowledge, this study is the first to examine the prevalence rates and associated demographic and clinical factors in individuals with previous unidentified BIF (IQ = 70–85) in both in- and out-patients receiving mainstream SUD services for polysubstance abuse. The current study's main findings are consistent with the few other studies from a SUD population, who identify an over-representation of impaired intellectual function among patients with SUD (24, 29). The current study adds on to these results by controlling for the effect of age, gender, years of education, age of onset of substance use, history of injecting drugs and satisfaction with life, in the analysis of the association between BIF and psychological distress.

The main limitation of this study concerns the representativeness of the Norwegian WASI test norms. Previous studies have shown that WASI tends to overestimate the FSIQ IQ level in Norwegian samples (92, 93), which may have led to the underestimation of the prevalence rate of BIF in the current study. In addition, the clinical differences between the BIF and non-BIF groups in the sample may have been masked if a skewed cut-of value of BIF have led to inclusion of non-BIF patients within the BIF group. Furthermore, WASI has not explicitly been validated for patients with SUD with a high level of psychological distress, which may also have affected the results of the present study. Finally, the STAYER cohort was recruited using convenience sampling in a clinical setting, which is vulnerable to ascertainment biases by undersampling patients with lower intellectual functioning, low motivation for change and lower-functioning patients with BIF.

### Clinical Implications

BIF among patients with SUD is common. Screening for intellectual functioning should therefore always be considered as part of the clinical practice, and treatment programs should account for a significant sub-population of patients with co-occuring SUD and intellectual impairments.

Clinicians should not only be wary of elevated levels of psychological distress in patients with SUD (54), but also that BIF may represent a potential added risk factor for detrimental treatment outcomes, drug-seeking behavior and relapse. Studies aimed at examining potential factors that mediate and moderate the relationship between psychological distress and intellectual functioning are therefore strongly warranted.

The current study could not establish a relationship between BIF status and social adjustment, which further highlights the importance of including data pertaining to everyday functioning in the assessment and diagnosis of ID, as well as the classification of borderline intellectual disability. Conjointly, measurements of general intellectual functioning may, to a lesser degree, predict social adjustment in patients with SUD. Furthermore, the associated risk factors as well as the long-term rehabilitation trajectories and prognosis of the co-occurrence of SUD and BIF are mostly unknown and warrant further investigation.

## Data Availability Statement

The raw data supporting the conclusions of this article will be made available by the authors, without undue reservation.

## Ethics Statement

The studies involving human participants were reviewed and approved by Regional Ethics Committee (REK 2011/1877). The patients/participants provided their written informed consent to participate in this study.

## Author Contributions

JH, EH, and AE: conceptualized and designed the study. JH: wrote the first draft and revised the manuscript. JH and AE: performed the analyses. AE, EH, KB, and AL: made critical revisions of the manuscript. AE and AL: supervised the study. All authors contributed to the article and approved the submitted version.

## Conflict of Interest

The authors declare that the research was conducted in the absence of any commercial or financial relationships that could be construed as a potential conflict of interest.
